# Development of di(2‐ethylhexyl) phthalate‐containing thioglycolic acid immobilized chitosan mucoadhesive gel as an alternative hormone therapy for menopausal syndrome

**DOI:** 10.1002/btm2.10267

**Published:** 2021-12-04

**Authors:** I‐Hsuan Yang, I‐En Lin, Ya‐Jyun Liang, Jhih‐Ni Lin, Tzu‐Chien Chen, Zhi‐Yu Chen, Che‐Yung Kuan, Chih‐Ying Chi, Chi‐Han Li, Hung‐Ming Wu, Feng‐Huei Lin

**Affiliations:** ^1^ Department of Biomedical Engineering College of Medicine and College of Engineering, National Taiwan University Taipei Taiwan; ^2^ Institute of Biomedical Engineering and Nanomedicine National Health Research Institutes Zhunan, Miaoli County Taiwan; ^3^ PhD Program in Tissue Engineering and Regenerative Medicine National Chung Hsing University Taichung Taiwan; ^4^ Department of Neurology Changhua Christian Hospital Changhua Taiwan

**Keywords:** di(2‐ethylhexyl) phthalate, menopausal syndrome, mucoadhesive gel, thioglycolic acid immobilized chitosan

## Abstract

Menopausal syndrome includes the symptoms that most women experience owing to hormone changes after menopause. Although hormone replacement therapy is a common treatment for menopausal syndrome, there are still many side effects and challenges hindering research. In this study, thioglycolic acid (TGA)‐immobilized chitosan mucoadhesive gel was synthesized by a new method of low concentration of 1,4‐butanediol diglycidyl ether (BDDE) would encapsulate di(2‐ethylhexyl) phthalate (DEHP) as an alternative hormone replacement therapy for menopausal syndrome. The efficacies of the DEHP‐containing TGA‐chitosan gel (CT‐D) were confirmed and evaluated by materials characterization and in vitro study. Results showed that CT‐D was not cytotoxic and had better mucoadhesive ability than chitosan. The animal model was constructed 1 month after bilateral ovariectomy in SD rats. CT‐D was administered intravaginally every 3 days. Bodyweight, wet weight of the uterus and vagina, vaginal smears, histology, blood element analysis, and serological analysis was used to assess the ability of the material to relieve menopausal syndrome. The results indicated that the combination of the sustained release of DEHP and mucoadhesive TGA‐immobilized chitosan allows the developed CT‐D to relieve the menopausal syndrome through low concentrations of DEHP, which falls in the safety level of the tolerable daily intake of DEHP.

## 1 INTRODUCTION

Menopause is the permanent suspension of the menstrual cycle due to a decline in ovarian function and changes in hormone concentration. During this period, estrogen levels decrease rapidly. Estrogen in women will drop from a normal or slightly high value to a low value (<30 pg/ml).^1^ Follicle‐stimulating hormones and the luteinizing hormone will also increase due to the lack of negative feedback from estrogen.^2^, ^3^, ^4^

Owing to hormonal changes, most women experience uncomfortable physical and mental symptoms, which is called menopausal syndrome. Hot flashes are a major symptom of menopausal syndrome. It will give women a sensation of instantaneous temperature rise spreading through the whole body and may be combined with sweating, chills, heart palpitations, and anxiety.^5^ Genitourinary syndrome is another common menopausal syndrome. The genitourinary tract of women undergoes a series of physiological and structural changes, including reduced vaginal rugae, decreased elasticity, loss of mature epithelial cells, and fibrosis of the epithelial tissue, which eventually causes vaginal atrophy.^6^ Other studies have shown that reduced estrogen may also reduce the metabolic rate, which leads to weight gain in some women after menopause.^7^

Hormone therapy is currently used to treat menopausal syndrome. Estrogen and combined hormone therapy are the most effective ways to treat moderate to severe menopausal syndrome. However, menopausal hormone therapies that are in clinical use are still doubtful with regard to their risks and benefits in preventing chronic diseases. Clinical reports indicate that the use of these hormones increase the risk of coronary artery disease, breast cancer, stroke, and venous thrombosis.^8^ Therefore, the current treatment recommendation is to use low‐dose estrogen to improve menopausal syndrome.

In addition to the hormone therapy mentioned above, there have been many studies on alternative therapies. The use of selective serotonin reuptake inhibitors, serotonin–norepinephrine reuptake inhibitors, and selective estrogen receptor (ER) modulators can improve hot flashes, anxiety, and benefit the maturation of vaginal epithelial cells. However, some side effects may occur, such as nausea, dry mouth, and constipation.^9^, ^10^ Other phytoestrogens, such as isoflavones, lignans, and coumestans as well as Chinese herbal medicines such as angelica, ginseng, ginkgo, and vitamin E are all potential alternative medicines, but their efficacies for menopausal syndrome vary for individuals. Some herbs are slightly toxic to the liver^11^, ^12^; therefore, hormone replacement therapy with lower side effects will provide a solution to the unmet needs of menopausal syndrome in clinical settings.

Di(2‐ethylhexyl) phthalate (DEHP) is a common plasticizer used in the polyvinyl chloride industry. At high doses, DEHP causes reproductive and developmental toxicity in animals.^13^ However, people are generally exposed to plasticizers daily with low concentrations of DEHP. Approximately 70%–90% of DEHP is excreted after 36 h by a single oral dose.^14^, ^15^, ^16^ More than 90% of the primary, secondary, and tertiary DEHP metabolites are rapidly excreted in the urine within 24 h postdose.^17^ Some researchers have also shown that DEHP at a low concentration of 10^−3^ mol/L could have estrogenic activities and stimulate the cell proliferation of human breast cancer Michigan Cancer Foundation‐7 (MCF‐7) cells in vitro.^18^, ^19^ In the vaginal delivery system, some polymers, such as hyaluronate, alginate, and methylcellulose^20^, ^21^ have been used in vagina atrophy; these polymers all have some limitations, for instance, leakage, short retention time, and lack of constant release in local delivery to the vagina. Conversely, the vaginal epithelium contains mucus layers, which can protect epithelial cells and prevent infection by microorganisms.^22^, ^23^ Because the mucus layers are composed of glycoproteins with cysteine‐, glutathione‐, and thioredoxin‐rich domains, thiolated polymers are promising materials that may form disulfide bonds with the mucus layers.^24^, ^25^ In previous studies, the phytoestrogen genistein was encapsulated in a 1,4‐butanediol diglycidyl ether (BDDE)‐crosslinked thioglycolic acid (TGA)‐immobilized chitosan gel. The oxidation–reduction reaction in the vagina tract could cause the materials to dissociate from the mucosa to prevent side effects of the excess dose residue.^26^

**SCHEME 1:**
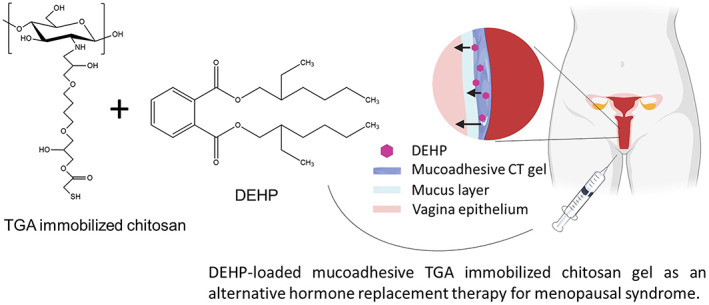
Scheme illustration of CT‐D preparation and its application for menopausal syndrome. Thioglycolic acid (TGA)‐immobilized chitosan was synthesized through 1,4‐butanediol diglycidyl ether (BDDE) crosslinking. Di(2‐ethylhexyl) phthalate (DEHP) was encapsulated in mucoadhesive TGA‐immobilized chitosan gel. A low‐dose DEHP and TGA‐immobilized chitosan gel would keep DEHP sustained‐release on the mucus layer for the long‐term treatment of menopausal syndrome

Presently, few studies have been conducted to evaluate DEHP to behave the estrogenic activities and be an alternative hormone replacement therapy for menopausal syndrome. In this study, we aimed to develop a DEHP‐containing TGA‐chitosan gel (CT‐D). A relatively low concentration of BDDE was used to crosslink and immobilize TGA onto the polymer chain. The thiol group on the materials could react with mucin to form disulfide bonds to achieve a constant release of DEHP from CT‐D for at least 3 days. We hypothesized that the combination of sustained‐release DEHP and mucoadhesive chitosan gel would allow CT‐D to relieve menopausal syndrome at a low concentration of DEHP, which falls in tolerable daily intake of DEHP. A low‐dose DEHP and TGA‐immobilized chitosan gel to keep DEHP‐containing chitosan sustained release on the mucus layer would be a new idea for the long‐term treatment of menopausal syndrome. The overall design is illustrated in Scheme 1.

The developed CT‐D was characterized by Fourier transform infrared spectroscopy (FTIR), Ellman's assay, ninhydrin test, and energy‐dispersive X‐ray spectroscopy (EDS) to identify the functional groups and the thiol contents of TGA‐immobilized chitosan. High‐performance liquid chromatography/tandem mass spectrometry (HPLC‐MS/MS) was used to determine the release profile of DEHP from the CT‐D. The Water‐Soluble Tetrazolium 1 (WST‐1) assay and Live/Dead staining were used to evaluate the cytotoxicity and cell viability of the CT‐D. The mucoadhesive ability was investigated using a microfluidic channel model composed of Caco‐2 cells and fluorescein isothiocyanate (FITC)‐labeled materials (CTF). The animal model was constructed after 1 month with the bilateral ovariectomy of Sprague‐Dawley (SD) rats. CT‐D was administered intravaginally every 3 days. Bodyweight, wet weight of the uterus and vagina, vaginal smear, hematoxylin and eosin (H&E), blood element analysis, and serological analysis were used to evaluate the efficacy of the materials.

## 2 RESULTS

### 2.1 FTIR analysis of CT

**FIGURE 1:**
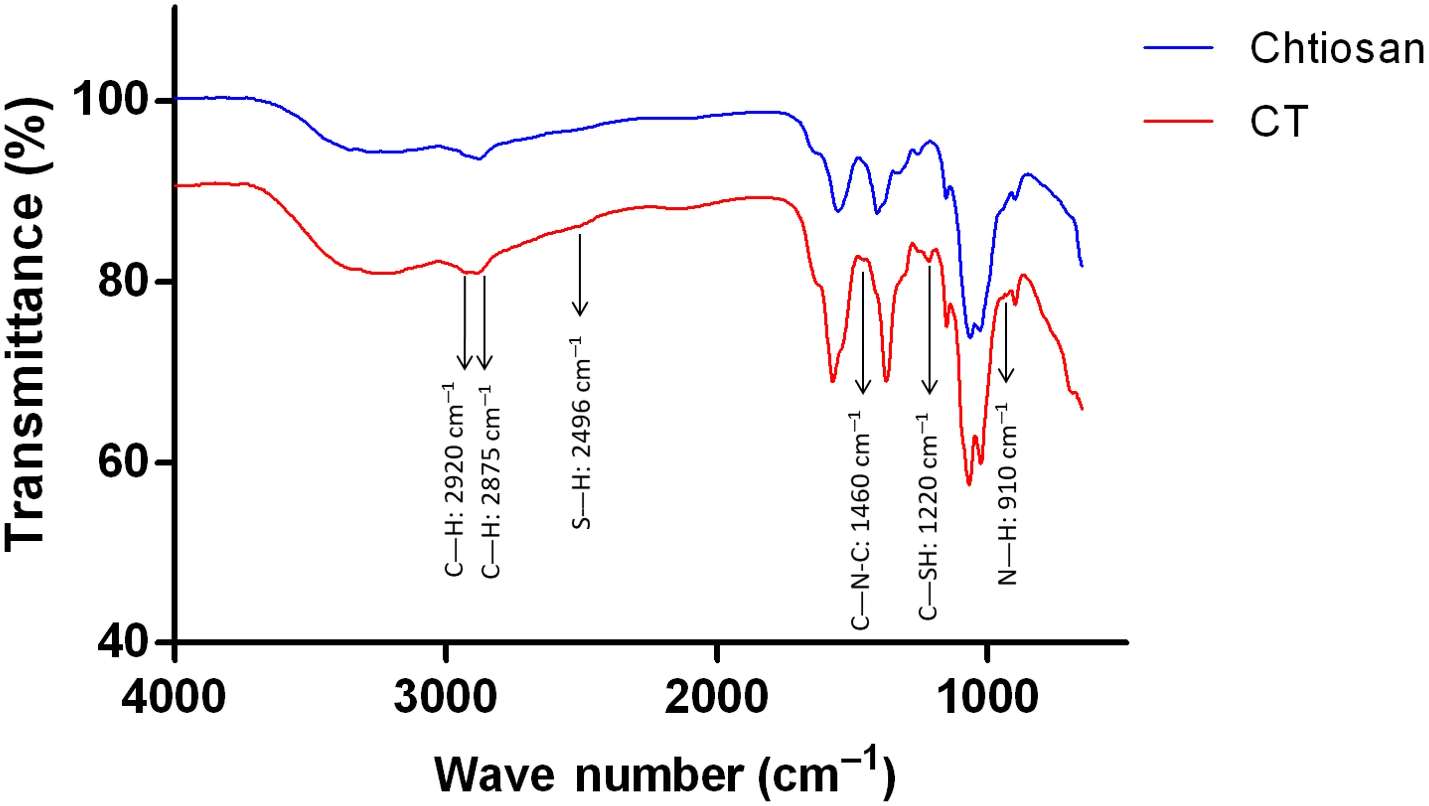
Fourier transform infrared spectroscopy (FTIR) of chitosan and thioglycolic acid (TGA)‐immobilized chitosan (CT)

To synthesize the CT, BDDE was used as the crosslinker to react the epoxy groups on BDDE with the amines on chitosan and carboxylic acid on TGA. The functional groups of the synthesized CT were analyzed using FTIR spectrophotometry. The spectra were recorded in the wavelength range of 400–4000 cm^−1^ (Figure 1). The absorption band at 2496 cm^−1^ corresponds to the stretching vibration of the thiol (─SH) group.^27^ The characteristic absorption band at 1220 cm^−1^ corresponds to the C—SH stretching, which was not observed in chitosan.^27^ The absorption bands at 1460 cm^−1^ and 910 cm^−1^ correspond to the C—N—C asymmetric stretching and N—H wagging vibration of the secondary amine, respectively.^28^ The bands at 2875 cm^−1^ and 2920 cm^−1^ were assigned to symmetric and asymmetric C—H vibrations, respectively, which were mainly due to the crosslinked BDDE molecule.^29^

### 2.2 The thiol content of CT

**FIGURE 2:**
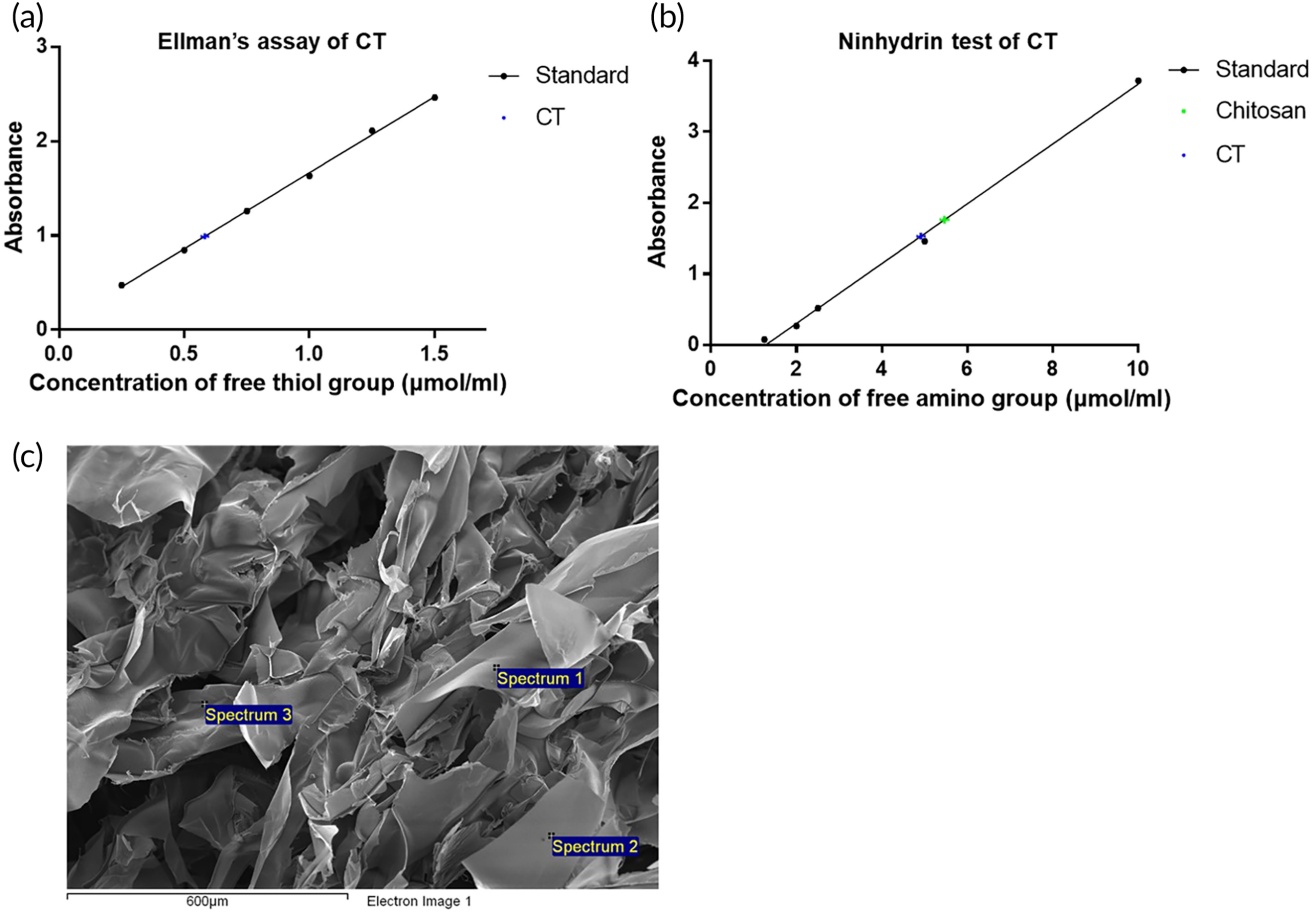
The thiol content of CT. (a) The thiol content of CT was tested by Ellman's assay. (b) The free amino group of chitosan and CT were evaluated by ninhydrin test. The reduced free amino groups in the synthesized CT indicated the thiols groups were substituted for the amino groups on CT. (c) Scanning electron microscopy images and the elemental analysis of the synthesized CT powder using energy‐dispersive X‐ray spectroscopy

The amount of free thiol groups on the synthesized CT was directly quantified by Ellman's assay, where the different concentration of cysteine was chosen as the standard. There were free thiols of approximately 0.58 ± 0.02 μmol/mg in CT, as shown in Figure 2a and summarized in Table 1.

**TABLE 1:** Summary of the Ellman's assay and ninhydrin test

	Amount of free thiol group	Amount of free amino group
Chitosan	ND	5.46 ± 0.10 μmol amino group/mg
CT	0.58 ± 0.02 μmol thiol group/mg	4.92 ± 0.09 μmol amino group/mg

Abbreviation: ND , not detected.

Ninhydrin test was further used to investigate the degree of grafting on CT. The free amino groups on the chitosan chain would react with the TGA through the BDDE crosslinking. There were free amino groups of approximately 5.46 ± 0.10 mM μmol/mg and 4.92 ± 0.09 μmol/mg in chitosan and CT, respectively. The reduction of amino groups was about 0.54 μmol/mg, as shown in Figure 2b and summarized in Table 1.

The EDS analysis was used to determine the chemical composition of the CT, as shown in Figure 2c. Three random points were chosen for elemental analysis. The results showed that there was approximately 6.61% sulfur in the CT, as summarized in Table 2. These results further proved that the TGA was successfully immobilized onto chitosan through BDDE crosslinking.

**TABLE 2:** Summary of the EDS analysis

CT	C	O	S
Spectrum 1	74.94	19.08	5.98
Spectrum 2	65.93	28.71	5.66
Spectrum 3	65.57	26.22	8.21
Mean	68.71	24.67	6.61

*Note*: All results in atomic %.

### 2.3 In vitro cytotoxicity of CT‐D

**FIGURE 3:**
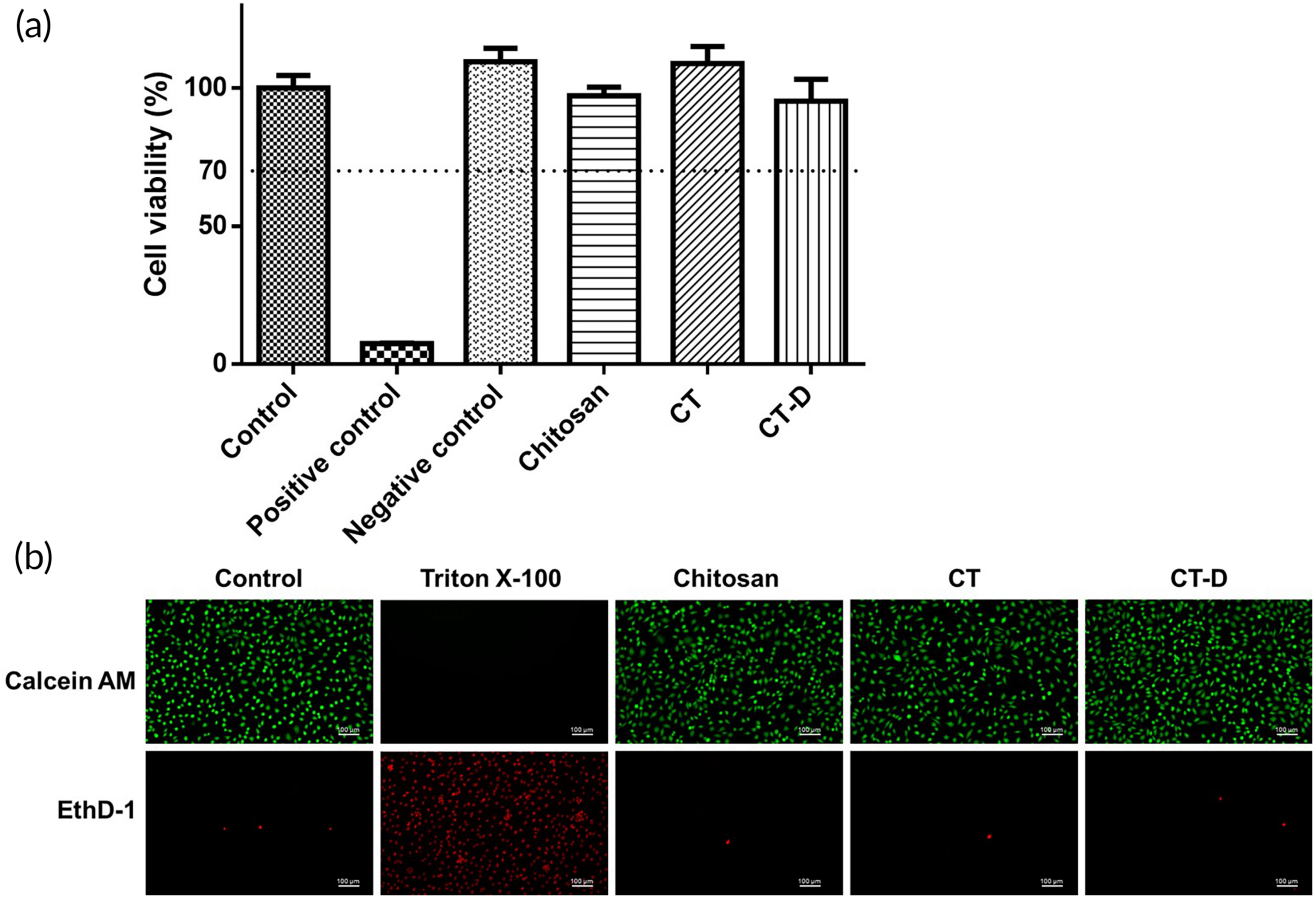
In vitro cytotoxicity of chitosan, CT, and CT‐D. (a) WST‐1 assay of chitosan, CT, and CT‐D were evaluated by L929 based on the guidance of ISO‐10993. All the groups had cell viability higher than 70%, showing that our materials did not have obvious cytotoxicity. (b) Live/Dead staining of chitosan, CT, and CT‐D. The live cells in green color were stained with calcein AM and dead cells in red color were stained with ethidium homodimer‐1 (EthD‐1). (Scale bar = 100 μm)

The in vitro cytotoxicity of the CT‐D was tested using the WST‐1 assay according to the International Organization for Standardization (ISO) 10993‐5 standard. The absorbance of WST‐1 at a wavelength at 450 nm was converted to cell viability. The results are shown in Figure 3a. The survival rate of all groups was higher than 70%, which means that the material had good biocompatibility and no significant toxicity to the target cells.

The CT‐D was further checked for cytotoxicity using the Live/Dead staining assay (Figure 3b). Cells cultured in a normal medium were used as the control group. Triton X‐100 treatment and extracts of chitosan, CT, and CT‐D were used as the positive control and experimental groups, respectively. The results showed that chitosan, CT, and CT‐D showed green fluorescence in living cells (Calcein AM), and the cell type did not change significantly. Only a few cells showed the red color of dead cells (EthD‐1), which shows that the material had no obvious cytotoxicity and had the same results as the WST‐1 assay.

### 2.4 DEHP release profile from CT‐D

**FIGURE 4:**
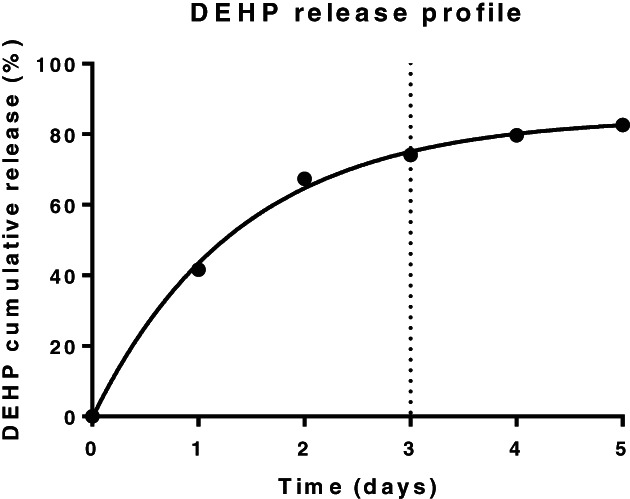
Release profiles of di(2‐ethylhexyl) phthalate (DEHP) from CT‐D. The cumulative DEHP released curve was measured by high‐performance liquid chromatography/tandem mass spectrometry (HPLC‐MS/MS)

Figure 4 shows the cumulative DEHP release profile. The release of DEHP from CT‐D was based on diffusion control. Approximately 41% of DEHP was released from CT‐D on the first day, and approximately 75% of cumulative DEHP was released on Day 3. The average 1 mg/ml/day of DEHP was released in the first 3 days, which is higher than the required concentration for estrogen activity.^18^ This result indicated that controlled release of CT‐D was achieved in this study, which was appropriate for the in vivo study.

### 2.5 Mucoadhesive property of CT‐D

**FIGURE 5:**
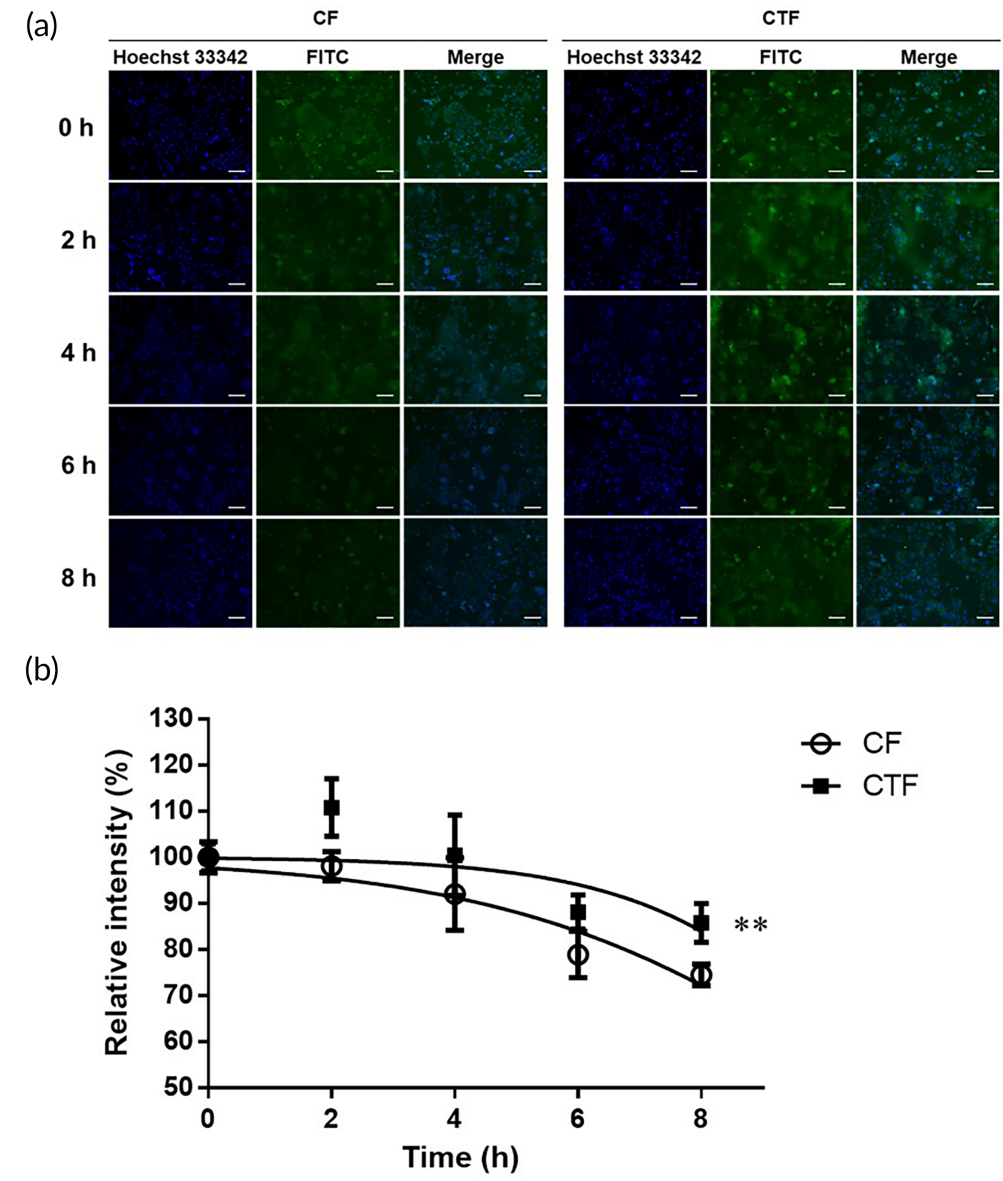
(a) In vitro mucoadhesive property was evaluated under a fluorescence microscope. The fluorescein isothiocyanate (FITC)‐labeled materials were shown in green, and nuclei were stained with Hoechst 33342, shown in blue. Scale bar = 100 μm. (b) Quantified percentage of the brightness of FITC. (**p < 0.01, compared to CF group)

Chitosan‐FITC (CF) and chitosan‐TGA‐FITC (CTF) were used in the in vitro mucus adhesion test. As shown in Figure 5a, the materials were first attached to the mucus layer and then perfused with the culture medium. Fluorescence images were taken at different perfusion times. The nuclei were stained with Hoechst 33342, shown as blue, where the FITC‐labeled materials are shown in green. In the beginning, there were still many FITC‐labeled materials in the medium, resulting in an obvious green background with the cells. Quantification of the FITC fluorescence image is shown in Figure 5b. The brightness at 0 h was set to 100%. As time progressed, the materials were washed away, whereas the CTF washed away slower than the CF. The results indicated that the thiols on the CTF could effectively conjugate to the mucus layer, thereby significantly enhancing the mucoadhesive property of the chitosan.

### 2.6 Bodyweight and wet weight of uterus and vagina

**FIGURE 6:**
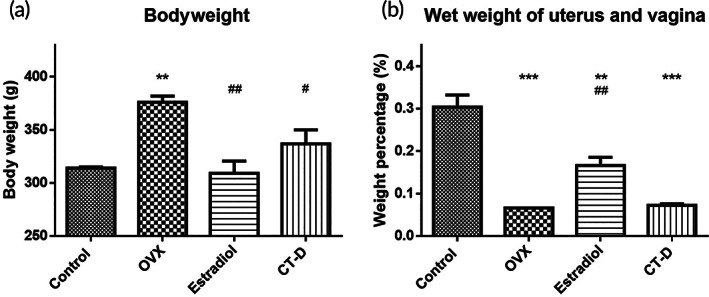
(a) Bodyweight of the rats of different groups and (b) wet weight of uterus and vagina of the rats in different groups. (**p < 0.01; ***p < 0.001, compared to control group; #*p* < 0.05; ##*p* < 0.01, compared to OVX group)

Figure 6 shows that the bodyweight of the ovary removal group (OVX group) significantly increased compared to that of the control group, which is consistent with previous studies.^30^ In contrast, when the administering estrogen ointment (estradiol group), the bodyweight significantly decreased compared with the OVX group. When CT‐D was administered to the rats, the bodyweight partially returned to normal as compared with the OVX group. Figure 6b shows that the weight of the uterus and vagina of the ovariectomized rats decreased significantly, whereas the weight in the estradiol group significantly increased, followed by the CT‐D group

### 2.7 Vagina smear

**FIGURE 7:**
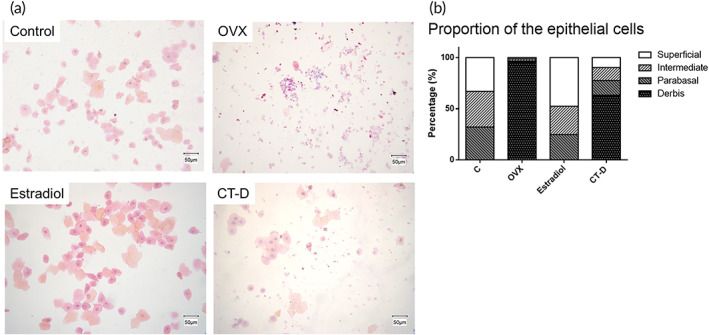
(a) Vaginal smear with Papanicolaou (PAP) staining. Scale bar = 50 μm. (b) Quantification of the proportion of the epithelial cells

The cell types in the vaginal smears can be represented by different vaginal maturities of the animal body. In general, a dominance of the superficial cells (the most mature type) and fewer parabasal cells (the immature type) was observed in the epithelium before menopause, indicating less vaginal maturities caused by menopausal syndrome (Figure 7a). The quantification of the cell types is shown in Figure 7b.

As shown in Figure 7a, the main cell types in normal rats were superficial cells with transparent nuclei and little cytoplasm, and intermediate cells with small nuclei; only a small number of parabasal cells and cell debris were observed in the control group. In the OVX group, the degree of vaginal maturity was low, whereas most cell types were cell debris and parabasal cells. The vaginal smears in the estradiol group showed a large number of superficial and intermediate cells, which were similar to the control group. Although there was still some cell debris, 37% were superficial cells, intermediate cells, and parabasal cells in the vaginal smears of rats administered CT‐D. These results indicated that CT‐D could partially recover vaginal maturity compared to ovariectomized rats.

### 2.8 Histological analysis of vagina tissue

**FIGURE 8:**
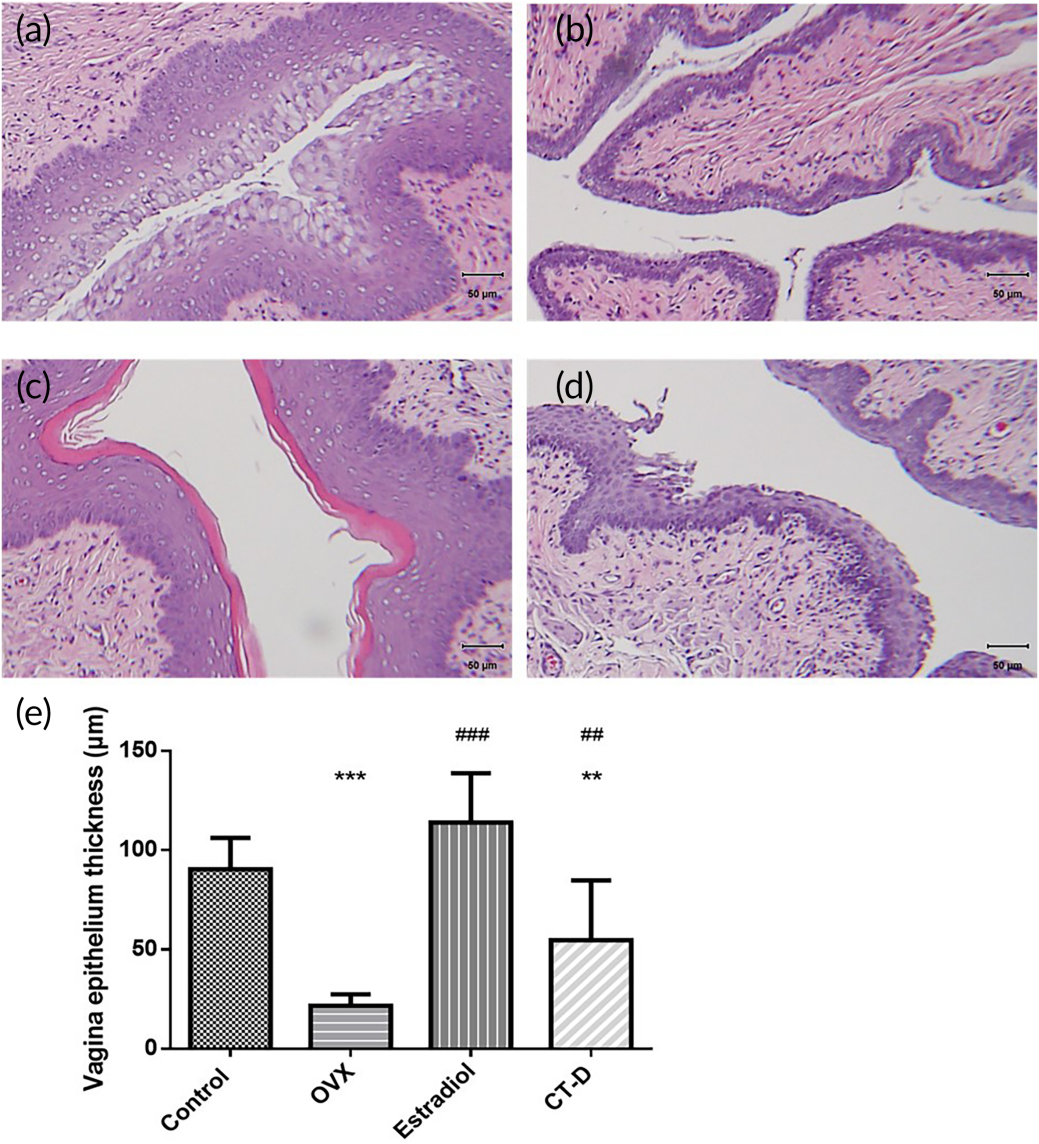
Hematoxylin and eosin (H&E) staining of the vagina (a) control group, (b) ovary removal group (OVX), (c) estrogen ointment group (estradiol), (d) CT‐D group. Scale bar = 50 μm. (e) Quantification of vaginal epithelium thickness of different groups. (**p < 0.01; *** *p* < 0.001compared to control group; ##*p* < 0.01; ###*p* < 0.001, compared to OVX group)

The effect of CT‐D on recovery of menopausal syndrome was evaluated by measuring vaginal thickness in the vaginal tissue sections with H&E staining (Figure 8a). The vagina thickness was measured at the boundary from the parabasal cell layers to the vagina surface (distance in the dark purple region), and the results are shown in Figure 8b. The vaginal layers were much thinner than the control group in ovariectomized rats due to a lack of estrogen. When the rats were administered estrogen ointment, the vagina thickness was significantly thicker than that in the OVX group. CT‐D also had a positive effect, which could effectively recover about 61% of the vagina thickness compared to the control group.

## 3 DISCUSSION

After menopause, women experience systemic or local discomfort, such as hot flashes, night sweats, insomnia, vaginal atrophy, dryness, and itching due to a sharp drop in the concentration of estrogen in the body.^31^ Currently, the prescription drugs used clinically are estradiol and its derivatives, but they may increase the risks of heart attack, stroke, and cancer due to endometrial hyperplasia.^32^ To reduce side effects, local administration methods have been adopted, such as the use of estrogen cream, tablets, or rings, but their effects on menopausal syndrome are very limited and require frequent dosage.^33^ In addition to the hormone therapy mentioned above, some people use water‐, silicone‐, or oil‐based moisturizers and lubricants to improve vaginal moisturization and relieve the discomfort of vaginal atrophy caused by menopausal syndrome.^34^ However, dyes, perfumes, and antibacterial agents in these products may be too irritating for the vagina. Their long‐term effects are also insignificant and can only be used to relieve symptoms.

To address these unmet clinical needs, in this study, CT loaded with DEHP was designed for intravaginal delivery. There are many advantages of intravaginal administration, such as a considerable surface area, rich blood flow, high degree of permeability to most of the drugs, in addition to avoiding the first‐pass effect of the drugs.^35^, ^36^, ^37^

BDDE, a crosslinking agent widely used in hyaluronic acid, cellulose, and chitosan,^38^, ^39^, ^40^ was used in this study to crosslink chitosan and simultaneously immobilize TGA onto the polymer chain. The thiol group content on CT was quantified by Ellman's assay, ninhydrin test, and EDS analysis, as presented in Figure 2 and summarized in Tables 1 and 2. The degree of grafting could be estimated by calculating the amount of the reduced free amino groups on chitosan and CT. In the ninhydrin test, about 0.54 μmol/mg of amino groups in chitosan were reacted with TGA through BDDE crosslinking, where the degree of grafting was approximately 10% (Table 1). The grafted TGA on the chitosan chain provided CT‐D with good mucoadhesive properties to the mucin (Figure 5).

Polymer gels loaded with drugs have been shown to achieve prolonged, steady drug release.^41^, ^42^, ^43^, ^44^ In the mucoadhesive property experiment, both the chitosan and CT were retained on the mucus layer in the first 2 h (Figure 5). When the medium was continuously perfused to mimic the vaginal environment, CT showed enhanced mucoadhesive properties compared to chitosan because CT could form covalent disulfide bonds with the mucus layer of the mucosal tissue to extend the retention time, which would enable the slow release of DEHP to avoid the self‐cleaning mechanism of the vagina.

DEHP is a man‐made chemical that is abundant in the environment. Numerous studies have shown that DEHP can interact with ERα and ERβ, acting on the endocrine system.^45^, ^46^ It has been shown that DEHP at a concentration of 10^−3^ mol/L could behave as estrogen‐like properties as 17β‐estradiol.^18^, ^19^ The toxic dosage of DEHP and the metabolic mechanisms in the body are also fully understood.^14^ The no‐observed‐adverse‐effect‐level (NOAEL) of DEHP was set at 4.8 mg/kg/day for multigenerational reproductive toxicity in rats.^17^ Moreover, the tolerable daily intake value of DEHP for the parenteral routes is 0.6 mg/kg/day for humans.^47^, ^48^ However, there is currently no research using DEHP to treat menopause syndrome. This study aims to develop a mucoadhesive TGA immobilized chitosan gel combined with the low‐dose DEHP to relieve the menopausal syndrome, while administering an effective dose within the daily tolerable intake of DEHP.

The obtained CT‐D gel showed good biocompatibility in vitro (Figure 3). CT could have a greater mucosal adhesion ability than unmodified chitosan (Figure 5). The sustained release of DEHP was achieved at a rate of 1 mg/ml for the first 3 days (Figure 4). In the in vivo study, an ovariectomized rat model was used to simulate the lack of estrogen secretion in animals as a result of menopause. Merely 0.5 mg/kg/day of DEHP in CT‐D was administered intravaginally in vivo for rats. According to the human equivalent dose calculation based on body surface area recommended by Food and Drug Administration (FDA), the human equivalent dosage of a 0.5 mg/kg rat is a 0.08 mg/kg human dose,^49^ which falls far below the safety level of NOAEL and tolerable daily intake of DEHP. The effects could be directly observed from the thickness of the vaginal epithelium and cell types in the vaginal smear. Before menopause, high levels of estrogen mature the vagina, generating thicker epithelial tissue and a large number of mature superficial cells. After menopause, the lack of estrogen causes the vaginal epithelium to become thinner, lack mature cells, and increase cell debris.^31^ When the rats were administered CT‐D, the vaginal epithelium thickness recovered to 61% of the control group, and the mature cells recovered to approximately 37% of the control group (Figures 7 and 8). We speculated reasonably that the sustained release of DEHP would interact with the ERs. The ER activity in the vagina epithelium is important for maintaining the proper structural integrity of the vagina.^50^ Hormone and growth factor signals, such as epidermal growth factor, keratinocyte growth factor, and insulin‐like growth factor‐1, would also be integrated by ER to mediate regulation of cell proliferation, vagina maturation, and thickness of the vagina epithelium.^51^, ^52^ Furthermore, other changes such as bodyweight and wet weight of the uterus and vagina also had a relative return to normalcy. In the blood element analysis (Table S1) and serological analysis (Table S2), most of the levels were in the normal range or consistent with the previous literature.^53^, ^54^ In addition, the materials can regularly excrete excess polymers and DEHP out of the body through the vagina's redox mechanism and cleaning mechanism. The results indicated that CT‐D showed no systemic toxicity and could be a potential treatment for menopausal individuals.

In this study, the efficacy of the developed CT‐D gel was determined by comparing it with that of commercial estrogen cream and ovariectomized rats. CT played an important role in the extension of DEHP release. DEHP release from CT‐D relieves menopausal syndrome. The results were confirmed both in vitro and in vivo. The concept of using low‐dose DEHP and CT gel to keep DEHP‐containing chitosan on the mucus layer and achieve sustained release in the local vagina is a novel concept in the treatment of menopausal syndrome. In the future, the CT‐D prepared in this study might deliver other biomolecules to treat menopausal syndrome or other symptoms caused by low estrogen levels.

## 4 CONCLUSION

In this study, we successfully developed a mucoadhesive DEHP‐containing TGA‐chitosan gel (CT‐D). The characterized functional groups and thiol content were identified and confirmed by FTIR, Ellman's assay, ninhydrin test, and EDS analysis, which confirmed that the thiol groups on the materials could react with mucin to form disulfide bonds to prolong the DEHP release behavior. The prepared CT‐D showed no cytotoxicity in vitro and no systemic toxicity in vivo. We proved that the synergetic combination of the sustained release of DEHP and mucoadhesive TGA‐immobilized chitosan could allow the developed CT‐D to relieve the menopausal syndrome in a low concentration of DEHP, which falls in the safety level of NOAEL and tolerable daily intake of DEHP. The mucosal thickness and mature cells in the CT‐D treatment group recovered to 61% and 37%, respectively, compared to the control groups. These findings fully support that CT‐D is novel and plausible for the treatment of menopausal syndrome.

## 5 MATERIALS AND METHODS

### 5.1 Materials

Chitosan (Cat. No. 448877, deacetylation degree 75%–85%, molecular weight 190–310 kDa based on viscosity measurement), 1,4‐BDDE, TGA, DEHP, Dulbecco's modified Eagle's medium (DMEM), minimum essential medium (MEM), and phosphate‐buffered saline (PBS) were purchased from Sigma‐Aldrich (St. Louis, MO, USA). The fluorescein isothiocyanate (FITC) isomer was purchased from Fluka (Switzerland). Fetal bovine serum was purchased from Hyclone (Logan, UT, USA).

### 5.2 Synthesis of TGA‐immobilized chitosan (CT)

The CT was synthesized as follows: TGA (0.37 ml) was added to 50 ml of 2% (w/v) chitosan solution in 0.1 M acetic acid and stirred at room temperature for 1 h. The premixed solution was added to the crosslinker, 0.098 ml BDDE in 50 ml of isopropyl alcohol (IPA), and stirred at room temperature for 3 h. The resultant solution was dialyzed against deionized water using a dialysis membrane (MWCO: 12,000 Da) for 4 days. The solution was freeze‐dried and stored at room temperature.

### 5.3 Modification of CT with FITC

The fluorescence‐labeled materials allowed observation under a fluorescence microscope for the mucus adhesion test. In this study, CF was synthesized as follows: 100 μl of FITC solution (2 mg/ml stock solution in IPA as solvent) was added to 50 ml of 2% (w/v) chitosan solution in 0.1 M acetic acid and stirred for 1 h in the dark. A total of 0.1 M NaOH was then added dropwise to the resultant solution to precipitate the CF. The CF was washed with deionized water thrice to remove the residual FITC. The TGA‐immobilized chitosan‐FITC (CTF) was synthesized as described in Section 5.2.

### 5.4 Preparation of DEHP‐containing CT gel (CT‐D)

For the preparation of the DEHP‐containing CT gel, DEHP was loaded into the CT gel as follows: A 3% (w/v) CT was prepared as in 0.1 M acetic acid. DEHP was then added to the CT to achieve a concentration of 4 mg/ml.

### 5.5 The analysis of FTIR

An FTIR spectrophotometer (Perkin Elmer, spotlight 200i) with an autoattenuated total reflectance (ATR) system was used to determine the functional groups of the CT. The detected wave number range was from 400 cm^−1^ to 4000 cm^−1^. The number of scans was 16, and the resolution was 4 cm^−1^.

### 5.6 The analysis of Ellman's assay

Ellman's assay was used to evaluate the free thiol groups in the synthesized CT. Briefly, Ellman's reagent solution was first prepared by dissolving 2.5 mg of 5,5'‐dithiobis‐(2‐nitrobenzoic acid) in 10 ml 0.1 M sodium phosphate buffer solution with a pH value of 8. The sample solution was prepared by dissolving freeze‐dried CT in 1 ml of 0.01 M of an acetic acid aqueous solution to achieve a concentration of 1 mg/ml. Subsequently, 800 μl of Ellman's reagent solution was mixed with 250 μl of the sample solution and incubated in the dark at 37°C for 15 min. The supernatant was measured on the ultraviolet (UV) spectrophotometer at 412 nm.

### 5.7 The analysis of ninhydrin test

Ninhydrin test was adopted to test the free amino group on the chitosan and CT and the procedure is briefly described in this section. Ninhydrin reagent solution was prepared as 3% (w/v) in 95% ethanol. The sample solution was prepared by dissolving 1 mg of chitosan or CT in 1 ml of 0.01 M of an acetic acid aqueous solution. Next, 0.5 ml of sample solution was then mixed with 0.25 ml of 2 M acetic acid and 0.25 ml of the prepared ninhydrin reagent solution and incubated in the dark at 70°C for 40 min. The supernatant was measured on the UV spectrophotometer at 570 nm.

### 5.8 The analysis of EDS

EDS (JEOL, JSM‐6510) was used to determine the chemical composition of the CT. The samples were examined using a scanning electron microscope at 10 kV, with the current being automatically adjusted.

### 5.9 In vitro cytotoxicity of CT‐D

The in vitro cytotoxicity of CT was evaluated using a WST‐1 assay and a Live/Dead staining assay. In the WST‐1 assay, L929 cells were seeded into 96 well plates at a density of 10,000 cells/well. MEM medium was used to extract zinc diethyldithiocarbamate (ZDEC) as a positive control and Al_2_O_3_ as a negative control. The medium was used to extract chitosan, CT, and CT‐D based on ISO 10993‐12 guideline, and these were used as the experimental group. After 1 day of extraction, the extract was centrifuged at 1500 rpm for 5 min, and the supernatant was filtered. The extract was added to the preseeded 96 plates for 1 day (*n* = 5), and the cell activity was measured via WST‐1 assay using an enzyme‐linked immunosorbent assay (ELISA) Reader to detect the absorbance wavelength at 450 nm.

In the Live/Dead staining assay, L929 cells were seeded into 24‐well plates at a density of 10,000 cells/well. Meanwhile, MEM medium was used to extract chitosan, CT, and CT‐D for 1 day. On the second day, the medium was replaced with the extract for another 1 day. Triton X‐100 was added as a positive control 5 min prior to staining in the positive control group. The medium extract was then removed and washed twice with PBS. Serum‐free MEM‐containing calcein AM (2 μM) and EthD‐1 (4 μM) was added to each well and incubated for 30 min. The plate was observed under a fluorescence microscope (Olympus FV10000).

### 5.10 Release profile of CT‐D

The release profile of CT‐D was tested using HPLC‐MS/MS. The CT‐D was placed in the transwells against deionized water. The deionized water in the outer chamber was replaced daily. Waters XBridge™ C18, 3.5 μm, and 4.6 × 150 mm columns were used in this study. The mobile phase of DEHP was 98% acetonitrile and 2% ddH_2_O, and the extraction time was approximately 10 min.

### 5.11 Mucoadhesive property of CT

To evaluate the mucoadhesive property of the synthesized CT, Caco‐2 cells, which could secrete mucosal layers such as vaginal epithelium, were chosen as the target cells. Caco‐2 cells were seeded in a commercial microfluidic (ibidi μ–Slide I ^0.4^ Luer) at a density of 50,000 cells/ml and attached overnight. Hoechst 33342 (1 μg/ml) was added to the channel to stain the nuclei of the cells for approximately 15 min. The CF or CTF (6 mg/ml) in a 0.1 M acetic acid solution was diluted 1:1 with the culture medium and replaced with the medium in the microfluidic for 1 h to allow the materials attached to the mucosal layers. The microfluidic was then observed under a fluorescence microscope. The microfluidic system was afterward perfused with the culture medium at a flow rate of 0.1 ml/min using a syringe pump to mimic the physiological environment of the vagina. Quantification of brightness was evaluated using ImageJ software.

### 5.12 In vivo study

A total of 24 Sprague–Dawley rats, 6 weeks old, were purchased from BioLASCO, Taiwan. The study protocol was approved by the Institutional Animal Care and Use Committee of the National Taiwan University (IACUC, No. NTU‐108‐EL‐00134).

The experiment was divided into four groups: rats with no treatment (control), bilateral ovariectomy with no treatment (OVX), given estrogen ointment (Estradiol) after OVX, and a DEHP‐containing TGA‐chitosan gel (CT‐D). The rats were given 150 μl of the respective material every 3 days. The rats were euthanized after the 1‐month treatment. Bodyweight, wet weight of the uterus and vagina, vaginal smears, histology, blood element analysis, and serological analysis inclusive of aspartate aminotransferase (AST), alanine aminotransferase (ALT), creatinine (CRE), and blood urea nitrogen (BUN) were used to assess the ability of the material to relieve vaginal atrophy.

### 5.13 Statistics

There were at least three replicates for each data point in this study and the values are expressed as “mean ± standard deviation.” The calibration curve with a known concentration has a linear correlation coefficient greater than 0.99. The statistical difference of the experiment was tested using a one‐way analysis of variance with multiple comparison tests. Differences were considered significant at a *p*‐value of less than 0.05, *p < 0.05, **p < 0.01, ***p < 0.001.

## CONFLICT OF INTEREST

The authors declare no conflict of interest.

## AUTHOR CONTRIBUTIONS

**I‐Hsuan Yang:** Formal analysis (equal); investigation (equal); methodology (equal); visualization (equal); writing – original draft (equal). **I‐En Lin:** Formal analysis (equal); investigation (equal); methodology (equal); visualization (equal); writing – original draft (equal). **Ya‐Jyun Liang:** Methodology (supporting); validation (equal). **Jhih‐Ni Lin:** Methodology (supporting); validation (equal). **Tzu‐Chien Chen:** Methodology (supporting). **Zhi‐Yu Chen:** Methodology (supporting). **Che‐Yung Kuan:** Methodology (supporting). **Chih‐Ying Chi:** Methodology (supporting). **Chi‐Han Li:** Methodology (supporting). **Hung‐Ming Wu:** Conceptualization (equal). **Feng‐Huei Lin:** Conceptualization (equal); formal analysis (equal); funding acquisition (lead); methodology (equal); writing – review and editing (lead).

### PEER REVIEW

The peer review history for this article is available at https://publons.com/publon/10.1002/btm2.10267.

## Supporting information


**Appendix** **S1**: Supporting information

## Data Availability

The data that supports the findings of this study are available in the supplementary material of this article
