# Supplementary material for: Development of di(2‐ethylhexyl) phthalate‐containing thioglycolic acid immobilized chitosan mucoadhesive gel as an alternative hormone therapy for menopausal syndrome
Source: Bioeng Transl Med. 2021 Dec 4;7(2):e10267. doi: 10.1002/btm2.10267 (PMC9115706; doi:10.1002/btm2.10267)
Supplement: Supplementary file 1 — Appendix S1: Supporting information [file BTM2-7-e10267-s001.docx]

Supporting information

**Development of di(2-ethylhexyl) phthalate-containing thioglycolic acid immobilized chitosan mucoadhesive gel as an alternative hormone therapy for menopausal syndrome**

I-Hsuan Yang^a,+^, I-En Lin^a,+^, Ya-Jyun Liang^a^, Jhih-Ni Lin^a^, Tzu-Chien Chen^a^, Zhi-Yu Chen^a^, Che-Yung Kuan^a,b^, Chih-Ying Chi^b,c^, Chi-Han Li^b,c^, Hung-Ming Wu^d^, Feng-Huei Lin^a,b,c,*^

**Affiliation:**

^a^ Department of Biomedical Engineering, College of Medicine and College of Engineering, National Taiwan University, No. 49, Fanglan Rd, Taipei 10672, Taiwan.

^b^ Institute of Biomedical Engineering and Nanomedicine, National Health Research Institutes, No. 35, Keyan Road, Zhunan, Miaoli County 35053, Taiwan.

^c^ PhD Program in Tissue Engineering and Regenerative Medicine, National Chung Hsing University, Taichung, Taiwan

^d^ Department of Neurology, Changhua Christian Hospital, No.135 Nanhsiao Street, Changhua 50006, Taiwan.

*^+^* I.-H. Yang and I.-E. Lin contributed equally to this work.

Corresponding Author

^*^ Feng-Huei Lin: E-mail: [double@ntu.edu.tw](mailto:double@ntu.edu.tw)

**Table S1** Blood element analysis

|  | Control | OVX | Estradiol | CT-D |
| --- | --- | --- | --- | --- |
| RBC (M/μL) | 7.78 | 7.87 | 8.08 | 8.94 |
| HGB (g/dL) | 14.2 | 14.7 | 15.3 | 16.1 |
| HCT (%) | 48.6 | 44.8 | 45.2 | 54.4 |
| MCV (fL) | 62.5 | 56.9 | 55.9 | 60.9 |
| MCH (pg) | 18.3 | 18.7 | 18.9 | 18 |
| MCHC (g/dL) | 29.2 | 32.8 | 33.8 | 29.6 |
| PLT (K/μL) | 1527 | 921 | 1057 | 1079 |
| WBC (K/μL) | 11.24 | 17.14 | 17.48 | 12.46 |
| NEUT (K/μL) | 1.38 | 1.44 | 0.88 | 0.81 |
| LYMPH (K/μL) | 9.33 | 14.31 | 15.87 | 10.97 |
| MONO (K/μL) | 0.42 | 1.01 | 0.53 | 0.53 |
| EO (K/μL) | 0.1 | 0.33 | 0.19 | 0.12 |
| BASO (K/μL) | 0.01 | 0.05 | 0.01 | 0.03 |
| RBC: red blood cell; HGB: hemoglobin; HCT: hematocrit; MCV: mean corpuscular volume: MCH: mean corpuscular hemoglobin; MCHC: mean corpuscular hemoglobin concentration; PLT: platelet; WBC: white blood cell; NEUT: neutrophil; LYMPTH: lymphocyte; MONO: monocyte; EO: eosinophil; BASO: basophil. | | | | |

**Table S2** Serological analysis

|  | Control | OVX | Estradiol | CT-D |
| --- | --- | --- | --- | --- |
| AST (U/L) | 96 | 255 | 92 | 204 |
| ALT (U/L) | 48 | 70 | 71 | 53 |
| CRE (mg/dL) | 0.5 | 0.4 | 0.4 | 0.5 |
| BUN (mg/dL) | 17 | 18 | 16 | 16 |
| ALT: alanine aminotransferase; AST: aspartate aminotransferase; Crea: creatinine; BUN: blood urea nitrogen. | | | | |
